# Castor Oil Tasteless

**Published:** 1913-09

**Authors:** 


Castor Oil Tasteless. Aromatic or tasteless Castor Oil is made as follows:

Saccharin .............................. gr. vii
Vanillin ............................... gr. ii
Oil of Cinnamon, Ceylon .................. gtt. viii
Oil of Anise ............................ gtt. vii
Alcohol ................................ ʒv
Castor Oil, to make ...................... ℥xxxii

